# We lived and breathed medicine - then life catches up: Medical students’ reflections

**DOI:** 10.1186/1472-6920-14-66

**Published:** 2014-04-01

**Authors:** Mia Hemborg Kristiansson, Margareta Troein, Annika Brorsson

**Affiliations:** 1 Department of Clinical Sciences, Lund University, Malmö, Family Medicine, Jan Waldenströmsgata 35, SE 205 02, Malmö, Sweden

## Abstract

**Background:**

Reflective writing enhances personal and professional development. It is essential for the teachers to be familiar with the students’ perceptions to improve the students’ learning. Our aim was to deepen the understanding of the medical students’ perceptions of the studies and the coming profession.

**Methods:**

Our theoretical perspective is constructivist, based upon the relativist view that individuals construct realities to understand and navigate the world. Constructivist methodologies are hermeneutic, with the focus on understanding rather than explaining. Thirty-five written reflections were collected in the first and fifth semesters at Lund University Medical School, Sweden. We used a thematic analysis, close to editing style analysis, inspired by K Malterud, who has modified Giorgi’s phenomenological method.

**Results:**

For first-semester students the focus is on studies and methods to structure them. The fifth semester is permeated by strategies for achieving a sense of ‘good enough’, qualities of a good doctor and applicability. Clinical placement as a motivating element is important for both semesters.

**Conclusions:**

A sense of ‘good enough’ is crucial for students to get by. Reflective writing can illuminate the strategies for achieving this. Clinical placement is vital for motivation.

## Background

Becoming a doctor is a process which consists of many different aspects. The development of professionalism is central, as pointed out in Medical professionalism in the new millennium: A physicians’ charter [1]. Reflection plays an essential role in professional training [2-5] and is included in the goals set for medical education [6]. Reflective writing is regarded as an effective tutorial tool in this process [2-4,7-12]. However, questions have been raised about whether we really know how these reflective assignments should be designed to have the best effect, and some authors stress the importance of feedback [8,10,13].

Medical students have different backgrounds, experiences, motives and expectations [7,9]. Therefore teachers can be helped in their planning of education if they are aware of the students’ views. This is also important in order to educate doctors who are well-functioning, satisfied with their profession and do not get burnout in the longer term [14].

Our aim in this study was to deepen the understanding of the medical students’ perceptions of their studies and their future profession, in order to enhance the learning environment for the students.

## Methods

### Theoretical approach

In this study our theoretical perspective is constructivist, based upon the relativist view that individuals construct realities to understand and navigate the world. In the constructivist perspective, the role of the researcher is subjective, that is, the researcher is also engaged in the construction of reality. Constructivist methodologies are hermeneutic and focus on understanding rather than explaining phenomena [15-17]. Reflective capacity is considered as a core of professional competence [2,4,5] and regarded as an important educational goal at most medical schools and other professional programmes [6,10,18]. Definitions of reflection within education originally derive from Schön and Dewey [5,19]. We chose to define reflection as a metacognitive process resulting in a greater understanding. The reflection links former experiences with future ones and affects actions and thoughts before, during and after a situation [20]. For the individual it is considered to enhance professionalism, which according to Schön in particular facilitates handling insecurity and ambiguity in professional practice [5]. Constant exposure to complicated situations in combination with structured reflection is crucial for optimal learning through studies and professional life [20].

### Data collection

Swedish medical education comprises eleven semesters (5.5 years). At Lund University Medical School professional development constitutes a recurring element during pre-clinical semesters 1–5, allowing the students to practise consultation skills, physical examination, ethics and legal knowledge. Medical students start with an introductory course. This gives an overview of the education and the profession, focusing primarily on early patient contact, the introduction of problem-based learning, ethics and professional reflection. As part of the examination all students write a reflection on their studies, and their views of the medical profession. This assignment is repeated during the fifth semester. The written reflections represent the basis for a professional, reflective discussion with a teacher. The instructions between the semesters differ slightly (see Appendix I).

In the autumn semester of 2011, all 118 first-semester students were invited to participate anonymously in this study with their written reflections and 16 choose to do so, 11 women and 5 men. Equally, all 115 fifth-semester students were invited, and 19 participated, 14 women and 5 men. Most texts consisted of 400–600 words. No extra work was needed other than to mail the reflection to the author MHK. All students received written and oral information including an assurance that participation in the study was voluntary and did not affect the examination. One reminder was mailed out 2–3 weeks after the initial information. When each reflection was registered it was anonymised, and content that could identify the writer was changed.

### Analysis

In this study we have used a thematic analysis inspired by K Malterud, who in turn has substantially modified Giorgi’s phenomenological method [16]. Malterud describes this method as being close to editing style analysis [21,22]. No selection was made since the total number of reflections was manageable, and all were included in the analysis. The reflections from semester 1 and 5 were initially treated separately. Each reflection was considered as a unit of analysis and read several times by MHK and ABR. The researchers did a parallel extraction of themes, and in some cases sub-themes, which turned out to be quite similar. A mutual, simultaneous condensation of codes and meaning units followed, which identified sections of the reflections addressing the initial themes and subthemes. Matrices were established, one for each theme, or one for each subtheme, if these appeared. Each code was placed as the heading of a column of meaning units. The principles for the condensation were to find a formulation encapsulating the meaning of the text sections, and that the sections should be neither too long nor too short (about two to four sentences). The matrices were revised by MHK and ABR and some codes were divided and others merged, some meaning units changing places by re-contextualisation. New matrices were established, visualising all meaning units, student by student. Major codes were selected by reflecting back on the aims of the study. The codes were revised in relation to the original texts, and most were found to be coherent and consistent between the researchers MHK and ABR. Eventually the matrices were examined regarding the age and sex of the writer.

### Ethics

Ethical approval was not needed according to the Ethical Review Board, Lund University, decision Number: 2010/384.

## Results

The predominant themes for both semesters 1 and 5 were motivation, perceptions of the medical education and profession, strategies for coping with the challenges as a student, and expected challenges in the future as a doctor. Semesters 1 and 5 will be presented separately and further comparison and consideration in relation to the sex and age of the writers will follow at the end of the results section.

### First-semester students

First-semester students focused on worries about the medical studies, learning for life and strategies for structuring life, and how to handle stress.

Almost half of the 16 first-semester students came to university directly from high school; only two of them had had other careers before going into medicine. Some reported work experience within healthcare, finding it self-reinforcing and useful in relation to their medical studies. Other issues highlighted from earlier life experiences were the ability to cope with people and to handle stress, knowledge of physically demanding jobs, or even of being unemployed. They also reported characteristics useful in their medical career such as ambition, curiosity, reflectiveness, helpfulness, and calmness, and an ability to be structured. In contrast to these characteristics, sensitivity to stress, insecurity, self-criticism and inflexibility were considered to be impediments.

For first-semester students thoughts about the medical education dominated over perceptions of the medical profession. The students wrote their reflection after only eight weeks of introduction, and the majority found the pace of the studies less stressful than they had expected. In general they were satisfied with their education at Lund University, appreciating problem-based learning (PBL), dedicated lecturers and the introductory course. Some were surprised by the variety of personalities and that not all students have parents who are doctors. Some worried about the future – and whether they would manage the increasing pace of studies they thought would come, and the difficult decisions they would have to make in their professional future.

Learning for life, not the examinations, was important for the motivation for the first-semester students. Many were also driven by an interest in biomedicine and by the variety within the medical profession. For some the single day with clinical placement they had had so far had increased their motivations for studying.

The major strategy to deal with the expected increasing demands was to structure the studies, not saving too much for the nights before an exam. A recurrent paradox was that some students perceive themselves as easy learners, entailing a sense of easy come, easy go, for example what is easily picked-up is easily forgotten, which they need to compensate for by finding new study routines. Being a quick learner was thus considered both positive and negative. Students suggested a variety of methods to cope with the stress: for one student, balancing between private life and studies was a way to handle it; another always put the studies first. For others the plan was to pass all exams the first time in order not to have the stress of re-examinations, while some in contrast would not take it too hard if they failed. Other methods were to work in healthcare during holidays, to reduce stress by being aware of it and to achieve better self-knowledge.

### Fifth-semester students

For the fifth-semester students the focus was on the essence of a good doctor and on how to achieve a sense of ‘good enough’. Other important topics were training of practical skills, enhancing motivation and giving a feeling of useful, applicable knowledge.

Almost a third of fifth-semester students mentioned their earlier life experience. Health care experience contributes to increased confidence and awareness in contact with patients. Curiosity and calmness were regarded as resources. Social capacity such as being a good listener, responsiveness in human interaction, humility and accountability were stressed. Negative sides were avoiding conflict, insecurity in oneself and one’s knowledge, and also carelessness.

‘A good doctor’ was a major code, which stated that a professional attitude is essential. Students’ views of professionalism included being proficient in biomedicine, almost considered as a self-evident requirement. On the other hand, the demand for knowledge was seen by some as infinite, leaving no room for mistakes since you are dealing with people’s lives. A capacity to synthesise theory and to combine practical knowledge with communicative and empathetic skill was believed to be even more significant. More than two thirds said that empathy was central. To have a true interest in other humans, reduce anxiety and be perceived as confident belonged to this empathic quality. Communicative skills found useful were being a good listener, perceiving the patient’s view, and taking into account the patients’ different backgrounds and medical knowledge. The synthesising capacity also included being open-minded in the diagnostic process, and practising clinical skills respectfully. Single students stressed the authoritarian, powerful part of the medical profession, and the difficult humane and economic priorities you need to make as a physician.

Motivation was important, but not sufficient, to acquire the huge amount of theoretical and practical knowledge that most students perceive as necessary to achieve. Study techniques such as a capability to sift out the most important facts, repetition, and structuring the studies as a full-time job, were some strategies for getting by. Discipline helped out when motivation was short, and so did finding a balance between leisure and studies: ‘The first semesters we lived and breathed medicine – but then life catches up with you’, an understanding that one needs time to recover. It was considered important to acquire a sense of what is ‘good enough’. This was achieved through strategies such as finding a balance between professional and personal life, letting go of perfectionism and handing in an assignment when it is finished even if not perfect and relying on the knowledge that the major issues recur several times during the medical training. Equally important in this struggle for ‘good enough’ was accepting that one cannot know everything and being honest about that and not being afraid to ask for help. One student could give herself an ‘OK seal’, and another student has accepted that he will be a clinician with an average knowledge of cell biology. As fifth-semester students they also noted that they perceived the work as a doctor more as a regular job and less as a vocation than they did in the early semesters. Heavy workload and responsibilities, substantial administration, and dissatisfaction among future colleagues were expected to be negative parts of their future profession.

The virtuous cycle of practical training of skills, clinical placements, and a sense of applicability were essential for fifth-semester students’ motivation. Meeting positive role models among practising doctors and getting feedback was also found stimulating. The professional education included most of these elements and was generally appreciated. It was also seen as self-reinforcing and important in personal and professional development. Some students expressed dreams about their professional future, almost as incantations, in the hope of becoming calmer with more practice: ‘not having shaky hands while pricking for a blood sample’, ‘not stopping reflecting, staying open-minded’, ‘being able to transmit security to the patients’.

### Comparison between semester 1 and 5

In the reflections of fifth-semester students a more humane attitude towards the medical profession and education dominated compared to the reflections of first-semester students. This included the ideal picture of a good doctor, the characteristics found useful in oneself, and the strategy of learning by doing. A clear difference between semesters 1 and 5 was the acceptance of a ‘good enough’ level of performance. Students in the fifth semester said that it is important to try to be ‘good enough’ and have the positive qualities of social competence and responsiveness, but this was hardly mentioned by first-semester students. First-semester students were more marked by a concern about how to manage at all. In semester 5 they felt the pressure, but also realised the impossible task of fulfilling all demands perfectly.

First-semester students instead stressed that it was helpful to be ambitious and structured. Calmness and curiosity were characteristics highlighted by students from both semesters, as well as the negative trait of insecurity. In both groups clinical placement was found to be a motivating element, although more clearly expressed in the fifth semester. This was naturally related to the difference in experience of the medical field between semesters 1 and 5.

A comparison considering possible differences between the content of male and female students did not show anything of interest, neither for each semester separately nor between the semesters. A majority of the students in both semesters were twenty-five years or younger. In relation to age there were likewise no significant differences except that the older students had broader life experience.

## Discussion

### Summary of the main findings

For first-semester students the focus is on the studies and more general expectations of the medical education. The central issues for the fifth-semester students are what constitutes a good, professional doctor, and how to feel ‘good enough’, as well as training of practical skills to enhance motivation.

### Comparison with existing literature

To pass through medical education implies not only learning knowledge but also developing a professional identity [23]. This is based upon the belief that identity is not something fixed that we are born with. “We construct and reconstruct our identities out of the various social cultural material” [24]. Hence the environment, both the open and the hidden curriculum at medical educational institutions, contributes in various ways to the process by which each individual medical student achieves a professional identity as a doctor [23]. Monrouxe points out the importance of understanding how this works and what implications it has for the design of medical education. Our material displays how different issues become important in various semesters and how the gradually increased experience combined with the varied content in the two semesters influence the students’ professional attitudes and reflections. Others have focused more on the problematic issues of this process – how an individual student can get squeezed in between his/her own convictions and the agenda of the medical institution [25]; how a non-coherent discourse from the institution causes problem for the students [24]. Our material, however, did not contain any of this kind of witnesses. There could be several reasons for this – that these issues were not problematic to our students, that too few reflections were collected to cover these points of view, or that the design of the assignment instructions did not stimulate this kind of reflections.

Professionalism includes the ability to deal with uncertainty [4,26,27]. Studies have been made on clinically working professionals’ strategies for dealing with uncertainty [28]. Other researchers have studied uncertainty amongst medical students, particularly fear of making mistakes, and found reflective writing to be an effective means for students to express and deal with uncertainty [11,29]. Those students, like ours, wrote after some time that they had tolerated themselves ‘as incomplete and […] as a good-enough doctor-to-be’ [11]. Unlike our results, however, they do not describe strategies for getting there. Our students point out various effective study techniques to leave space for private life. Other strategies are to consciously let go of perfectionism, to be honest about lack of knowledge, and to dare to ask for help when needed. Their strategies could fit into the coping theories of Folkman and Lazarus, with the first example being a problem-focused way of coping and the others being emotion-focused forms [30]. The finding in our study is opposed to other studies which have found students displaying strong defences against criticism and denial of uncertainty [26], and strategies for acclimatising to a professional rhetoric which allows display of some sides of uncertainty but not others [31]. It has even been shown how low tolerance for uncertainty amongst first-year students in fact predicts an excessive reliance on high-technology, a negative orientation towards psychological problems amongst the patients and a Machiavellianism [32]. In studies among working professionals, developing a sense of good enough has been found to be essential for working with efficiency and endurance [33]. Another aspect of ‘good enough’ is satisfaction with one’s own performance and confidence. It is claimed that working physicians whose fathers are physicians and those who have a high level of perceived clinical skills are most satisfied with their work [34]. This is related to general self-esteem. Some have found personality traits among medical students to be predictors of future stress and depression while working as junior physicians [35]. Finset et al. also considered gender differences, finding these two issues more important for females than males. Others have found gender differences in the content of written professional reflections [36], with female students being less confident, more concerned with balance between private and professional life. None of this is found in our material, which may be due to the small number of reflections in our study compared to the other studies.

Ever since Osler at the beginning of the 20th century, clinical education has been important at medical schools [37]. Clinical placement as early patient contact [38], as well as good role models among supervising senior doctors, are confirmed as motivational for medical students [2,7]. Early meaningful contact with patients is also believed to give an opportunity for reflection and integration of learning and help in the transition from student to working doctor [12,29]. Some of this is reinforced with the accounts primarily from our fifth-semester students. In our case some reported deepened theoretical knowledge from clinical reality; that clinical placement clarified how much there is left to learn and what the knowledge is needed for. This is coherent with Kolb’s “experiental learning cycle” [39]. The learning goes through four main phases – experiencing, reflecting, thinking, and acting. In the first phase, the learner has an experience. Reflection follows as a second phase which leads to abstract conceptualisation, the third phase. This is a very important phase where understanding of the previous actions and reactions is crucial. Application of the new knowledge and skills takes place in the fourth phase. This cycle can be repeated several times with “increased learning through each cycle”. Reflective writing can facilitate this process. Various educational tools can be used and portfolios often include both documentation and reflective writing [13]. Researchers claim that reflective writing is useful, for example to enhance learning and personal and professional development [2,7-10,12]. How to design these exercises to be most efficient is a matter of debate, however. Wald declares that reflective capacity is known to be ‘associated with improved diagnostic reasoning […] such direct correlation to RW [reflective writing] does not presently exist.’ [8]. Others state the importance of integrating reflective assignments as portfolios with the curriculum of the education and the benefits of combining it with feedback from tutors [10,13]. McGuire et al. [10] claim that detailed instructions facilitate the reflective writing process, but without showing how. It has not been one of our aims to study this, but we have a sense of the opposite. We have not found research evaluating the best ways to enhance reflective capacity for medical students, and thus the advantages of reflective writing or reflective discussions. This is an important research field.

### Strengths and limitations

Within qualitative research, validation can be addressed as trustworthiness [17,39]. This study fulfilled several criteria for trustworthiness as suggested by Graneheim, Lundman [17] and based on Lincoln and Guba [40]: credibility, dependability and transferability. Credibility consists e.g. of choosing an appropriate method, context and selection. Thematic analysis influenced by phenomenology is a method for exploring experiences and life world within a certain field [16,41]. It helps us to find the essences and important features in the subject, in this case medical students’ perceptions of their studies and future profession. We found this form of analysis suitable to grasp these issues which correspond to the aims of the study. It was also found to be adequate in relation to the amount of material we dealt with. We also found the context and the choice of units of analysis, one reflection for each medical student, relevant for the aims of the study. Editing style analysis was chosen for its ability to open for new and unexpected themes during the analysis.

The participation was fairly low in both semesters, in total 15%. The reasons for this are not obvious. Despite the information that participation in the study would not affect the results or the examination, the students might still have believed it would. Interviews could have given a deeper confidence in us as researchers. Logistic matters could also have played a part. When the reminder was mailed out proportionally more students mailed in their reflection than when the appeal was first handed out on paper. Nevertheless, for a qualitative study the number of participants is not as vital as for a quantitative one. In relation to credibility the richness and descriptive values are more important. We assume that the students who chose to participate have a special interest in reflection on professional matters. This presumably implies that we were provided with the richest material and hence this fulfils the intentions of purposive sampling, which is desirable within qualitative research [42]. On the other hand, it may give a positive bias in their judgement of the professional education. The content of the reflections may also have been influenced by the fact that they were a part of an examination, making them write what they thought would benefit them. Some of the differences between students in semesters one and five may be related to the different wording of the instructions for their assignments. The instructions for the first semester are more detailed and closed, e.g. a direct question about how they react to stress as a medical student. More open questions similar to those for the fifth semester would probably have generated other answers, as would of course interviews, which are often used in a phenomenological approach [42].

Two researchers working parallel and together in the analysis process constitutes another aspect of credibility. Here we stress the value of dialogue more than consensus in every respect [17]. All three researchers participate as tutors and teachers in professional development and one (MT) is the head of the course. This might be contradictory to having an open mind within the field, but prior knowledge of the structure of the education could be seen as vital and enriching when reading the students’ reflections. Using written reflections as material also give the study good dependability with a collection of material limited in time and amount [17,40] We hope the reader will find that the presentation of the context, data collection and description of the analysis process contributes to the transferability of the study.

## Conclusions

The students’ strategies for achieving a sense of ‘good enough’ are vital for them to manage their studies. Some of these strategies could presumably also be useful for a working physician as well. Reflective writing is one way to highlight these strategies, and pointing them out is regarded as useful within education in professional development. How instructions for assignments in reflective writing need to be designed to best stimulate reflective capacity needs to be further investigated, as well as the outcome of reflective discussions with a tutor. Clinical placement supports students’ learning and increases the pre-clinical students’ motivation.

Knowledge about the students’ strategies to achieve a sense of ‘good enough’ is helpful and important when structuring medical education and instructions for reflective assignments. In revision of the education it can also stress the need for practical training of various areas since the students identify this as one of the most effective ways of learning skills.

## Appendix I

Writing instructions for the assignment on semester 1: ‘Have you studied anything else before medical school? Do you have work experience – if so, as what? Have your studies so far influenced your picture of medical studies? How do you react to stress in your new situation as a student in the medical programme? Reflect on both strengths and weaknesses within yourself. What do you particularly need to develop? How do you plan to work with your studies? What are your personal goals?’

Writing instructions for the assignment in semester 5: ‘Reflect on your view of the medical profession when you wrote your last reflection – what has changed during these five semesters of studies? Focus on the knowledge, skills, and attitudes you think a doctor should have. What have you achieved in each of these fields? What are your strong and weak personality traits, in relation to your studies and in relation to your future profession? How would you like to work with or improve the weaknesses?’

## Competing interests

The authors declare that they have no competing interests.

## Authors’ contributions

AB and MT planned the study. The data collection was mainly done by MHK. AB and MHK performed the analysis. MHK drafted the manuscript, and ABR and MT participated in the process of editing the text. All authors read and approved the final manuscript.

## Authors’ information

Mia Hemborg Kristiansson is a registered physician, now training to become a specialist in general practice. She has worked as a radio journalist for the Swedish national radio and has a BA in literature. Margareta Troein is an MD and PhD in family medicine/general practice. She is a professor at Lund University, responsible for the course Professional Development during the first five semesters of medical school. Annika Brorsson is an MD and PhD in general practice/family medicine. She works part-time with teaching, supervising and research at Lund University.

## Pre-publication history

The pre-publication history for this paper can be accessed here:

http://www.biomedcentral.com/1472-6920/14/66/prepub
